# Evaluation of drug delivery vehicles for improved transduction of oncolytic adenoviruses in solid tumor tissue

**DOI:** 10.48101/ujms.v130.11217

**Published:** 2025-01-27

**Authors:** Erik Yngve, Sofie Ingvast, Olle Korsgren, Di Yu

**Affiliations:** Department of Immunology, Genetics and Pathology, Uppsala University, Uppsala, Sweden

**Keywords:** Co-drug, transduction efficacy, oncolytic virus, hyaluronidase, collagenase, polycations, DEAE-dextran, protamine sulfate, branched PEI

## Abstract

**Background:**

Oncolytic viruses are promising tools for immune stimulatory gene therapy of cancer, but their clinical effect on solid tumors have so far been limited. Transduction of the target tumor cells is limited by both extracellular matrix that blocks viral spread within the solid tumor tissue and electrostatic forces that inhibit virus from binding its entry receptor on the cell surface. The enzymes *hyaluronidase* and *collagenase* and the polycations diethylaminoethyl (*DEAE)-dextran*, *branched Polyethylenimine (PEI)* and *protamine sulfate* have previously shown potential to improve gene transfer in different forms of viral gene therapy, since they may help the virus to overcome these barriers. In this study, we compared the transduction-enhancing potential of these substances when used as vehicles for adenoviral transduction in solid tumor tissue.

**Methods:**

Subcutaneous tumors of pancreatic ductal adenocarcinoma were established in mice and treated with a mix of adenoviral vector Adf35(GFP-Luc) and either one of the selected vehicles. Transduction efficacy was determined by quantification of the viral transgene expression level using live imaging.

**Results:**

Addition of hyaluronidase tripled the transgene expression of Adf35(GFP-Luc) when compared to virus alone. No such positive effect was seen for the other tested vehicles.

**Conclusions:**

Out of the tested candidates, hyaluronidase showed the best potential to facilitate viral spread in tumor tissue and transduction of tumor cells. Therefore, hyaluronidase may be used as vehicle to improve clinical efficacy of oncolytic virotherapies.

## Introduction

Oncolytic viruses are promising tools for treatment of cancer but have not yet reached a breakthrough in clinical treatment regimens, mainly due to inefficient transduction of tumor cells. After intratumoral administration, which until today have been the most successful route of administration, the virus is trapped in a limited area close to the injection site and needle tract (1, 2). High intratumoral pressure and dense extracellular matrix (ECM) limit diffusion and convection of the injected virus as well as cell-to-cell mediated spread of progeny virus (1, 2). Additionally, repelling electrostatic forces between negatively charged viral- and cellular surfaces limit entry of virus into tumor cells (3).

To overcome these barriers, co-injection of oncolytic virus mixed with enzymes degrading ECM or polycations neutralizing repellant electrostatic charges have been proposed (4, 5). Examples of such agents are the enzymes *hyaluronidase* and *collagenase* and the polycations *DEAE-dextran*, *branched PEI* and *protamine sulfate*. They have previously shown potential as drug delivery vehicles in viral gene therapy by increasing the efficacy of transduction *in vivo* (3, 4, 6–11). Additionally, with exception for Branched PEI, they have been evaluated with favorable safety profiles in clinical trials, as vehicle for locally administered chemotherapy (hyaluronidase) (12), for treatment of Dupuytren’s contracture (13) and uterine fibroids (collagenase) (14), hypercholesterolemia (DEAE dextran) (15) and reversal of heparin-induced anticoagulation (protamine sulfate) (16). Thus, if effective, these substances, here referred to as vehicles, could efficiently be used to facilitate the transduction of oncolytic viruses in clinical settings.

Pancreatic ductal adenocarcinoma (PDAC) is a severe form of cancer with poor prognosis and, in many cases, no effective treatments are available. The PDAC microenvironment is characterised by dense ECM and few infiltrating tumor reactive cytotoxic T-cells. These are reasons why conventional chemotherapy and immunotherapies are not yet effective against this disease. Oncolytic viruses have the potential to stimulate anti-tumor immunity in PDAC but in order to make such therapy successful, transduction efficacy of the administered viral particles needs to be improved.

Although *hyaluronidase*, *collagenase*, *DEAE-dextran*, *protamine sulfate* and *branched PEI* are known to improve transduction in vector-based gene therapy, it is yet unknown if these substances are effective in facilitating transduction of oncolytic adenoviruses in solid tumor tissue.

In this study we compared the transfection enhancing potential of these vehicles *in vitro* and *in vivo*. Adenoviral vector Adf35(GFP-Luc) was mixed with different vehicles and injected in PDAC tumors established in mice. Transduction was measured through viral transgene expression levels. Hyaluronidase significantly improved the transduction, supporting its potential as vehicle in oncolytic virotherapy of solid tumors.

## Material and methods

### Cell lines and culture conditions

The human embryonic retinoblast E1A complemented cell line 911, human PDAC cell line Panc-01 and the mouse PDAC cell line Panc-02 were cultured in complete culture media containing Dulbecco’s modified Eagle’s medium (DMEM) with 10% heat-inactivated fetal bovine serum (FBS), 100 IU/mL penicillin, 100 μg/mL streptomycin and 1 mM sodium pyruvate. Cell cultures were maintained in 95% humidity and 5% CO_2_ at 37°C.

## Recombinant adenoviruses

The adenoviral vector Adf35(GFP-Luc) was used *in vitro* and *in vivo* to study its transduction efficacy when administered with different vehicles. Adf35(GFP-Luc) is an adenoviral vector of serotype 5 with the capsid fiber generated from adenovirus serotype 35, binding to human cellular receptor CD46, which often is abundantly expressed on tumor cells (17). The vector is non-replicating due to a full deletion of the E1 gene, which is replaced by the genes encoding green fluorescent protein (GFP) and Luciferase (Luc). This vector was used since it allows easy readout of viral transgene expression through luciferase-based assays and imaging techniques.

The oncolytic adenovirus Adf35(OGN) was used *in vitro* to further study the effect of hyaluronidase, since that vehicle showed promising results with Adf35(GFP-Luc). Adf35(OGN) is a conditionally replicating oncolytic adenovirus with a 24 base pairs deletion in the E1A gene and replacement of the whole E1B gene with genes encoding alpha-1,3-galactosyltransferase and neutrophils activating protein, which are expressed in infected cells to stimulate anti-tumor immunity. Like Ad5(GFP-Luc), Adf35(OGN) is a human adenovirus of serotype 5 with the capsid fiber generated from adenovirus serotype 35. Adf35(OGN) is currently undergoing preclinical evaluation with the aim for further evaluation in clinical trials.

Recombinant adenoviruses were generated by λ-phage-mediated recombineering in *E. coli* strain SW102 as described previously (18, 19). Viruses were produced in cell line 911 and and purified through cesium-chloride ultracentrifugation and dialysis as described previously (20).

The functional viral titers were determined by a fluorescent forming unit (FFU) assay on 911 cells, as described previously (21). Viral genome copy numbers were determined by quantitative PCR with specific primers detecting the viral genome region E4 ORF1. Plasmid, pCR2.1(AdE4orf1) containing the same amplicon, was used for the generation of standard curve, as described previously (22). Viral titers are presented in Table 1.

**Table 1:** Viruses used in this study. Total and infective viral titers are presented.

Virus	Total viral titer (encapsidated viral genomes (evg)/mL)	Infective viral titer (FFU/mL)	evg/FFU
Adf35(GFP-Luc)	1.1 × 10^12^	2.5 × 10^11^	4
Adf35(OGN)	6.6 × 10^12^	1.7 × 10^11^	40

### Animals

Animal experiments were performed as approved by the regional ethics committee (d.nr. 5.8.18-19434/2019). Six weeks old immunocompetent female C57Bl/6N and -/6J mice were purchased from the breeders Taconic and Charles River, respectively, and were housed at the Rudbeck animal facility (Uppsala, Sweden) in individually ventilated cages with five mice per cage.

### Vehicles

The following vehicles; *Hyaluronidase*, c*ollagenase*, *DEAE-dextran*, *protamine sulfate* and *branched PEI* were selected for evaluation in this study since they previously have shown potential to improve viral transduction *in vivo* (3, 4, 6–11). Data from three of these previous studies formed the basis for calculating estimated optimal vehicle concentrations (EOC) to be used in this study. Each vehicle, supplier, EOC and reference study is listed in Table 2.

**Table 2:** Vehicles evaluated in this study. Suppliers and estimated optimal concentrations for 50 μL injection volume (EOC) are presented. In addition, reference publications, reported administrational routs, vehicle doses, injection volumes and concentrations are presented.

Vehicle	Supplier and details	Estimated optimal concentration (EOC)	Reference publication	Reference adm. route	Reference vehicle dose	Reference Injection/instillation volume (μL)	Reference vehicle concentration
Hyaluronidase	Ref. H3506. Merck. Bovine. 700 U/mg	1 U/μL	Ganesh et. al. 2008 (9)	Intratumoral injection	50 U	50	1 U/μL
Collagenase HA	Ref. 001-1000 VitaCyte	0.1 μg/μL	McKee et. al. 2006 (4)	Intratumoral injection	1 μg	10	0.1 μg/μL
DEAE-dextran	Ref. 93556. Merck	20 ng/μL	Kaplan et. al. 1998 (3)	Intranasal instillation in lung	914 ng	50	20 ng/μL
Branched PEI	Ref. 408727. Merck	1 ng/μL	Kaplan et. al. 1998 (3)	Intranasal instillation in lung	46 ng	50	1 ng/μL
Protamine sulfate	Ref. P4020. Merck	10 ng/μL	Kaplan et. al. 1998 (3)	Intranasal instillation in lung	585 ng	50	10 ng/μL

### Transduction efficiency in vitro

Before the vehicles were tested in animal experiments, different concentrations were tested in viral transduction of cell cultures of human pancreatic cancer cells. This was done to identify any negative impact of the vehicle on the viral transduction and to further refine vehicle concentrations.

Panc-01 cells were harvested, distributed in 96 well plates and incubated over night for plate adhesion. The culture media was replaced by 100 uL transduction media containing complete culture media, virus and vehicles. Virus was added in a dilution series with multiplicity of infection (MOI) ranging from 5 to 625 FFU/cell. Vehicle concentrations ranged from 0.024 to 10xEOC (hyaluronidase), 0.024 to 100xEOC (collagenase and protamine sulfate) or 0.024 to 500xEOC (DEAE dextran and Branched PEI) (Supplementary Figure S1). The total volume was 100 μL per well and the infected cells were incubated at 37°C for 48 h.

**Figure 1:**
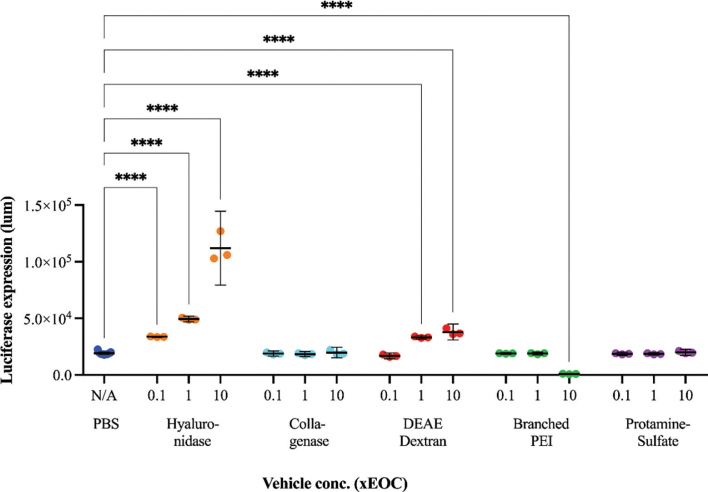
Transduction efficacy indicated by luciferase expression (y-axis) after *in vitro* transduction of Panc-01 cells with Ad5(GFP-Luc) in combination with vehicles (x-axis). Individual technical replicates, mean and 95% CI are shown. Each vehicle was tested at three different concentrations 0.1, 1 and 10 times EOC (Table 2). Individual groups were compared to the ‘virus alone’ control group using one-way ANOVA and Dunnett’s multiple comparisons test. ****P ≤ 0.0001.

When suitable viral dose (MOI) and concentrations for the respective vehicles were found, the experiment was repeated to directly compare the vehicles to each other. MOI of 50 FFU/cell and vehicle concentrations of 0.1, 1 and 10 times EOC were used (Figure 1) based on the previous results.

Since hyaluronidase showed improved transduction *in vivo*, the combination of hyaluronidase and the oncolytic virus Adf35(OGN) were tested *in vitro*. Hyaluronidase concentrations of 1 and 10xEOC was tested, combined with Adf35(OGN) at MOI 5 FFU/cell and, as reference, Adf35(GFP-Luc) at MOI 50 FFU/cell.

To measure transgene expression of luciferase *in vitro*, substrate from ONE-Glo™ Luciferase Assay System (Promega Biotech AB, Sweden) was added directly to the cell culture media in proportion 1:2 (substrate to culture media). Luminescence was measured using BioTek Synergy HTX Multimode Reader (Agilent). Transgene expression of GFP was measured by flow cytometry. Transgene expression of α-gal in cells infected with Adf35(OGN) was measured by flow cytometry after staining with Alexa Fluor™ 594 conjugated Isolectin GS-IB_4_ (Invitrogen). Readout was done using CytoFLEX LX Flow cytometer (Beckman Coulter). The flow cytometry results were analyzed using FlowJo™ v10.10 Software (BD Life Sciences). Standard gating was applied to select live single cells (FSC-H vs. FSC-A followed by FSC-A vs. SSC-A) for further analysis of GFP-expression in FITC-channel and α-gal expression in PE-channel.

To evaluate if hyaluronidase could also improve viral cytopathic effect of oncolytic virus, Panc01 cells were plated in 96 well plates (7 × 10^4^ cells per well) and the culture media was supplemented with hyaluronidase at different concentrations (Figure 4E) and Adf35(OGN) at MOI 1.4 FFU/cell. After five days incubation cell viability was measured by Alamar Blue assay (Invitrogen) according to the manufacturer’s instructions. Excitation/emission was analyzed using CLARIOstar Plus plate reader (BMG Labtech).

### Transduction efficiency in vivo

*In vivo* experiments in mouse were performed to study the transduction efficacy of the viral vector Adf35(GFP-Luc) mixed with either one of the vehicles (*hyaluronidase*, *collagenase*, *DEAE-dextran*, *protamine sulfate* and *branched PEI*) in solid PDAC tumors. Tumors were established subcutaneously on the hind flank through injection of 5 × 10^5^ mouse PDAC tumor cells (Panc-02) suspended in 100 μL PBS.

When tumors of around 150 mm^3^ were formed, Ad5(GFP-Luc) and vehicle was injected intratumorally at a viral dose of 1 × 10^9^ FFU per injection/ in a total volume of 50 μL.

To determine transduction efficacy, the transgene expression of luciferase was measured 24 h after virus injection. Mice were injected subcutaneously with 150 mg/kg d-luciferin (PerkinElmer), diluted in PBS. Ten minutes after the d-luciferin injection, mice were imaged using an IVIS Lumina II (PerkinElmer) bioluminescence imaging apparatus under isoflurane gas anesthesia. Image analysis and quantitation of the luciferase expression (total flux, photons/s) were done using Living Image software (version 4.7.1, PerkinElmer).

### Anti-adenoviral immunofluorescent staining in tumor tissue

After live imaging, mice were euthanized and tumors were collected. Snap frozen tumor tissue was sectioned (10 μm) and stained with anti-adenovirus antibody clone 20/11 (Sigma-Aldrich) directly conjugated with Alexa fluor 647 using Molecular Probes antibody labelling kit (By Life Technology) and nuclear staining. The stained sections were examined by Zeiss LSM700 confocal microscope.

### Statistics

Statistical analysis for all experiments was performed in GraphPad Prism 10.3.1 (GraphPad, La Jolla, CA, USA). When analyzing technical replicates normal distribution and equal standard deviation (SD) were assumed. When analyzing biological replicates, the Shapiro-Wilk test was used to analyze normal distribution. If significant (data not normally distributed) for any group included in the analysis a non-parametric test was used. Otherwise, parametric tests were used and equal SDs were assumed. If several experimental groups were compared with one experimental reference group, one-way ANOVA with Dunnett’s multiple comparisons test or Kruskal–Wallis test with Dunn’s multiple comparisons test was used. If more than two experimental groups were compared, one-way ANOVA with Tukey’s multiple comparisons test or Kruskal–Wallis test with Dunn’s multiple comparisons test was used. A *P*-value of less or equal to 0.05 was considered statistically significant. The *P*-values were represented as; *P ≤ 0.05, **P ≤ 0.01, ***P ≤ 0.001, ****P ≤ 0.0001.

## Results

### Estimated optimal vehicle concentrations for experiments in vitro and in vivo

The selection of vehicles and estimation of suitable concentrations for initial experiments was based on previously reported similar studies and is described in detail in the material and methods section. Vehicles and EOC are presented in Table 2. When vehicle concentrations are presented in this study they are presented as multiplicities of the EOC.

### Supplementation of hyaluronidase and DEAE-dextran in the growth medium improves transduction of human pancreatic tumor cells in culture

In order to optimize the transduction conditions, human pancreatic tumor cells (Panc-01) were infected with Adf35(GFP-Luc) at different MOI in the presence of different vehicles at different concentrations around EOC (Supplementary Figure S1). In brief, we observed that transgene expression was proportional to the MOI, and different vehicles had different effect on transduction efficacy. For further *in vitro* experiments, MOI was fixed to 50 FFU/cell and the vehicles were tested at concentrations ranging from 0.1 to 10 × EOC (Figure 1).

Hyaluronidase improved transduction in a concentration dependent manner up to the highest tested concentration 10 × EOC (Figure 1).

Collagenase did not affect transduction in the dose range of 0.1 to 10 × EOC (Figure 1). At 100 × EOC transduction increased slightly, especially at low MOI (Figure S1).

DEAE-dextran improved transduction with a peak in transduction efficacy around 1–10 × EOC (Figure 1 and Supplementary Figure S1). Branched PEI showed no effect at 0.1 to 1 × EOC but impaired transduction at 10 × EOC (Figure 1 and Supplementary Figure S1). Protamine Sulfate did not show any effect, neither positive nor negative, on the transduction in the concentration range 0.1 to 10x EOC (Figure 1) but increased transduction at 100x EOC (Supplementary Figure S1).

### Hyaluronidase improves transduction in solid tumor tissue

**Figure 2:**
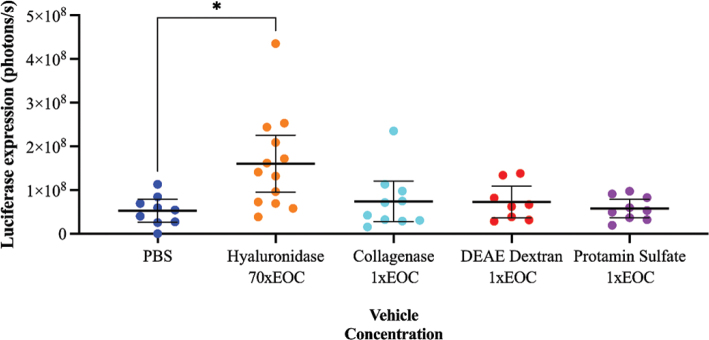
Transduction efficacy indicated by luciferase expression (y-axis) measured by *in vivo* imaging system (IVIS) 24 h after transduction of solid tumor tissue with Ad5(GFP-Luc) in combination with vehicles (x-axis). Individual biological replicates (*n* = 9–13), mean and 95% CI are shown. Each group was compared to all other groups using Kruskal–Wallis test and Dunn’s multiple comparisons test. *P ≤ 0.05.

**Figure 3:**
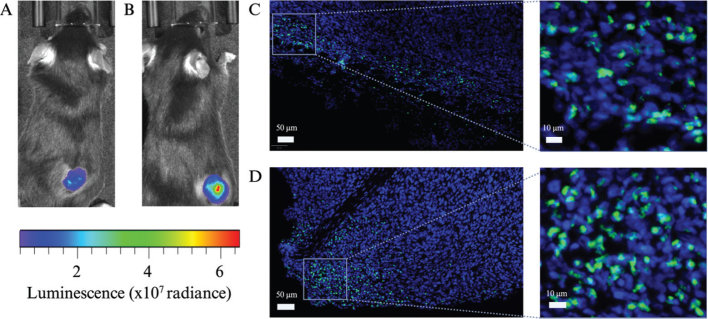
Representative images of Panc-02 tumors injected with Adf35(GFP-Luc) alone (A and C) and with hyaluronidase as vehicle (B and D). Transgene expression was detected by IVIS (A and B) and distribution of viral particles in the tumor tissue was depicted by immunofluorescent anti-andenovirus staining (green) and nuclear staining (blue) (C and D).

The vehicles were further tested in solid tumors in mice. Hyaluronidase did not show any positive impact on the transduction at one and seven times EOC (Supplementary Figure S2) but was well tolerated. Therefore, 70xEOC was tested and at that concentration median and mean luciferase expression tripled compared with virus alone (*P* = 0.02) (Figure 2 and 3A and B). Viral particles were also detected with a patchy distribution within the tumor tissue, with virus located in the cytoplasm (Figure 3C and D).

Collagenase was initially tested at 100xEOC, since that was the only concentration that showed a tendency to improve transduction *in vitro*. That dose was not tolerated *in vivo* due to severe tissue digestion and hemorrhage, which exceeded the tumor borders. The maximal tolerated dose was determined to 1x EOC and was used in further investigation, but showed no improvement of viral transduction (Figure 2).

DEAE-dextran and protamine sulfate did not affect transduction *in vivo* (Figure 2) at 1xEOC.

Animals treated with Branched PEI showed a substantial variation in transgene expression (Supplementary Figure S2). Considering that branched PEI is not approved for clinical use and its negative impact on transduction *in vitro*, this product was not included in further investigation.

In summary, supplement of high dose hyaluronidase to Adf35(OGN) enhanced the transgene expression in solid tumor tissue. No significant enhancements were observed for the other tested vehicles in this setting.

### Hyaluronidase improve transduction efficacy and cytopathic effect of oncolytic virus Adf35(OGN) in human pancreatic tumor cells

**Figure 4:**
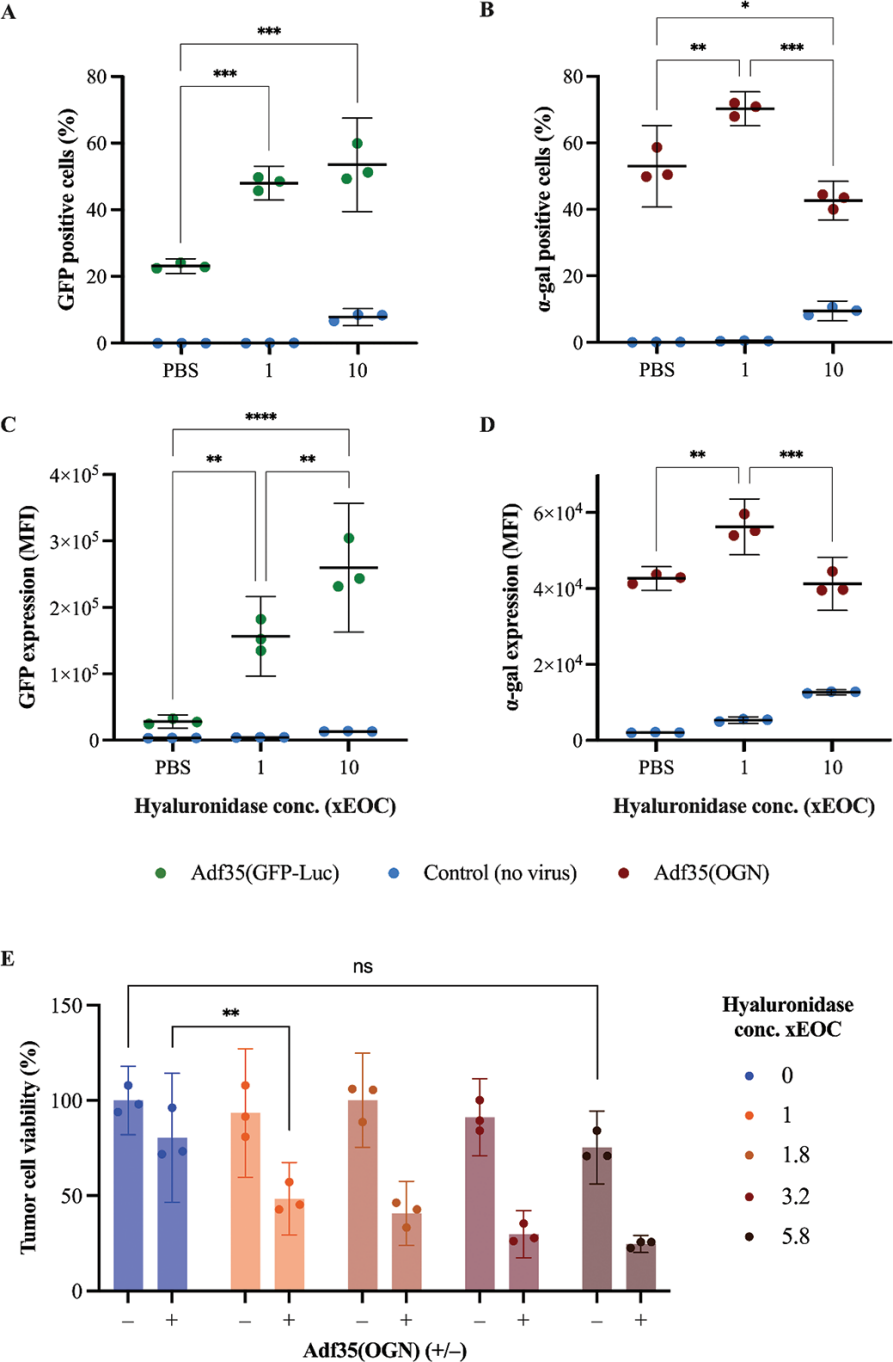
Hyaluronidase effect on transduction and cytopathic effect with Adf35(OGN). Panc-01 one cells were infected with the viral vector Adf35(GFP-Luc) (A and C) or the oncolytic virus Adf35(OGN) (B and D). Uninfected cells served as negative control (blue dots) for virus induced signal. The vehicle hyaluronidase was supplemented to the growth medium at two different concentrations, 1x and 10x EOC (x-axis). PBS served as negative control for vehicle induced signal. Transgenic GFP and α-gal was detected by flow cytometry, and percentage of cells expressing transgenes (A and B) and mean fluorescence intensity (MFI) (C and D) were calculated from viable single cells. Panc01 cells were treated with various concentrations of hyaluronidase ranging from 0 to 5.8 xEOC and either non-infected or infected with Adf35(OGN) at MOI 1.4 FFU/cell (E). After 5 days in culture the cell viability was analyzed by Alamar blue assay and relative viability compared to the non-hyaluronidase, non-virus control (left blue bar) was calculated (y-axis). Individual technical replicates, mean and 95% CI are shown. Groups were compared using one way ANOVA and Tukey’s test for multiple comparisons. No statistics are shown for uninfected control groups (A-D). *P ≤ 0.05, **P ≤ 0.01, ***P ≤ 0.001, ****P ≤ 0.0001.

Since hyaluronidase showed promising results with Adf35(GFP-Luc) both *in vitro* and *in vivo*, its potential as vehicle was further tested *in vitro* using a Adf35(GFP-Luc) and a therapeutic oncolytic adenovirus, Adf35(OGN), expressing a new combination of immune stimulatory transgenes. Transduction efficacy was evaluated by transgene expression of GFP and α-gal, respectively, and measured by FACS. As previously observed, hyaluronidase increased transduction efficacy of Adf35(GFP-Luc) in a concentration dependent manner (Figure 4A and C). When combined with Adf35(OGN) hyaluronidase increased transduction efficacy at 1xEOC but reduced it at 10xEOC. Among viable single cells, the proportion of α-gal positive cells increased from 53 to 70% at 1xEOC, but was reduced to 43% at 10xEOC (Figure 4B). A corresponding pattern was also found in mean fluorescent intensity from the same population (Figure 4D).

In order to test whether hyaluronidase improves the cytopathic effect of Adf35(OGN), cells were cultured with virus and different concentrations of hyaluronidase. After five days, cell viability was measured (Figure 4E). Hyaluronidase, at 1xEOC, significantly increased the viral cytopathic effect. Higher concentrations of hyaluronidase seemed to further increase the viral cytopathic effect in a dose dependent manner, although not statistically significant. In the uninfected controls, hyaluronidase did not affect tumor cell viability in the tested range (1 to 5.8xEOC).

## Discussion

In this study, we have shown that hyaluronidase is a potentially important vehicle for improving viral transduction in solid tumor tissue. Hyaluronidase tripled transduction efficacy *in vivo* whereas no statistically significant improvements were found for collagenase, DEAE-dextran, protamine sulfate and branched PEI.

In addition, hyaluronidase improved viral transduction *in vitro* for Adf35(OGN), which is an oncolytic adenovirus intended for use in clinical trials.

In solid tumor tissue, hyaluronidase likely degrade ECM and by that facilitate viral spread from the injection site and in turn increase viral access to tumor cells. This is supported by the clearly enhanced transduction efficacy.

It is not clear how hyaluronidase improve transduction *in vitro*. Potentially tumor cells in culture may produce some hyaluronic acid that form a physical barrier for viral infection, which is reduced by the supplement of hyaluronidase in the culture medium. Degradation of other cell surface molecules may also facilitate contact between virus and cell surface entry receptors. Additionally, altered plate adhesion and cell–cell contacts may increase the cells’ metabolic activity and thus increase transgene expression and viral replication, further leading to increased viral cytopathic effect. This interpretation is supported by a previous study in which embryonic fibroblast proliferation was stimulated by hyaluronidase *in vitro* (23).

When combined with the non-replicating vector Adf35(GFP-Luc), hyaluronidase improved viral transduction and therefore increase transgene expression in a dose dependent manner. However, when combined with the oncolytic virus Adf35(OGN), hyaluronidase seems to reduce transgene expression at 10xEOC. This is likely because high concentration of hyaluronidase, combined with a replicating oncolytic virus, rapidly cause viral cell death and thus reduce transgene expression.

Collagenase did not show the beneficial effect reported previously (4), using the same enzyme concentration. At higher concentration the treatment was not tolerated due to tissue damage and bleeding. Thus, potential severe side effects and narrow therapeutic window imply that collagenase may not be suitable as vehicle in oncolytic virotherapy.

We were not able to verify the previously reported beneficial effect of polycations used in cell culture studies (6, 24–26) or in *in vivo* transduction of healthy lung tissue (3, 10). Both models differ from the solid tumor tissue in the sense that cells susceptible to transduction are much more accessible. Lanuti et al. showed improved surface transduction of solid intraperitoneal tumors when adenovirus was administered with protamine sulfate, but viral penetration into the tumor tissue was still limited (11). Thus, the lack of efficacy for polycations in this study may reflect the entrapment of virus locally at the injection site implying that access to tumor cells is the primary limiting factor rather than repellant electrostatic forces that prevent viral contact with entry receptors. It is possible that polycations would be more effective in a less dense tissue. Therefore, the combination of hyaluronidase and polycations may be considered in future studies.

This study is limited to evaluate the transduction of tumor cells during the initial round of infection, upon viral injection. Even though, this is likely representative also for the therapeutic effect of an oncolytic virus, the effect of hyaluronidase on tumor development and survival in oncolytic virotherapy remains to be investigated. In such a setting, the potential immune stimulatory effect of hyaluronidase and fractionated hyaluronic acid (27), may complement the effect of increased viral transduction.

In conclusion, among the evaluated candidates, hyaluronidase clearly stands out with promising capacity to potentiate transduction and cytopathic effect of oncolytic adenoviruses. Transduction is a critical aspect of oncolytic virotherapy that fundamentally impacts viral oncolysis and immune stimulation. Thus, the addition of hyaluronidase to oncolytic virotherapies may increase therapeutic efficacy and limit treatment related toxicity.

## Supplementary Material



## Data Availability

Data are accessible upon a sensible inquiry.
