# Supplementary material for: Evaluation of drug delivery vehicles for improved transduction of oncolytic adenoviruses in solid tumor tissue
Source: Ups J Med Sci. 2025 Jan 27;130:10.48101/ujms.v130.11217. doi: 10.48101/ujms.v130.11217 (PMC11836772; doi:10.48101/ujms.v130.11217)
Supplement: Supplementary file 1 [file UJMS-130-11217-s1.pdf]

Supplementary materials

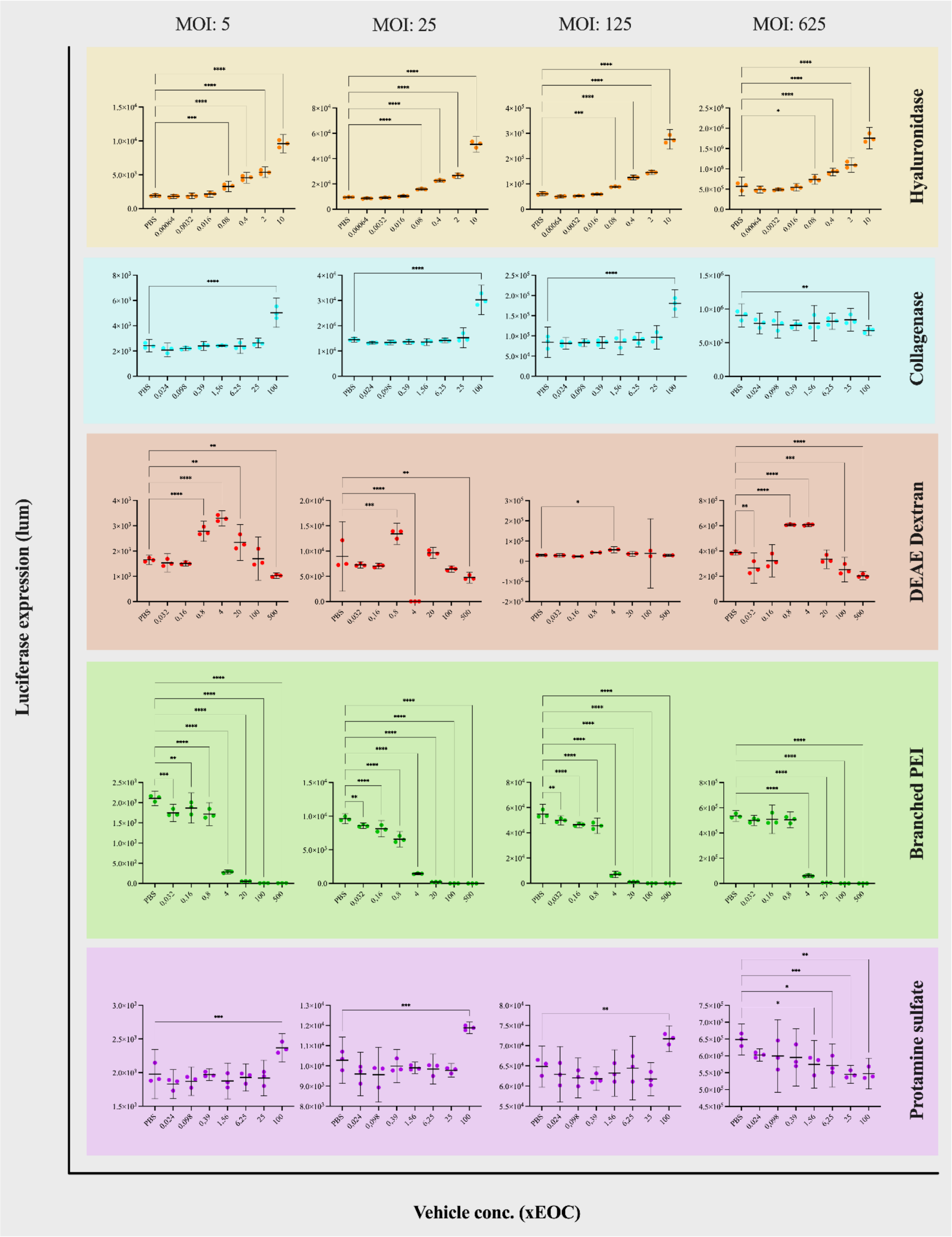

Supplementary figure S1. Transduction efficacy indicated by luciferase expression (y-axis) after *in vitro* transduction of Panc-01 cells with Ad5(GFP-Luc) in combination with vehicles. Note that range of the y-axis is adjusted to fit the data of each graph. Individual technical

replicates, mean and 95 % confidence interval are shown. Each vehicle was tested at seven different concentrations (x-axis) where the numbers indicate multiplicity of estimated optimal concentration (EOC). Individual groups were compared to the PBS control group using one-way ANOVA with Dunnett's multiple comparisons test. *$p \leq 0.05$, **$p \leq 0.01$, ***$p \leq 0.001$, ****$p \leq 0.0001$.

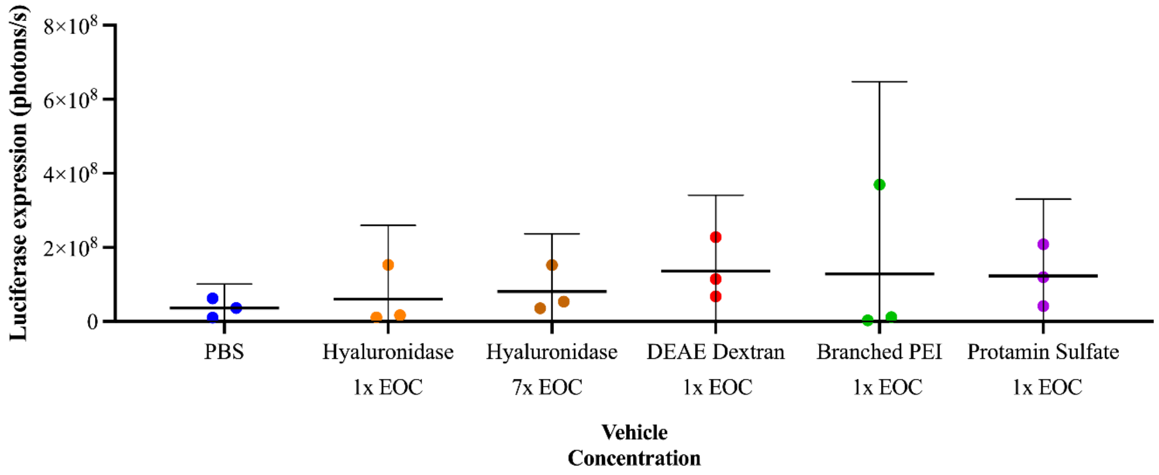

Supplementary figure S2. Transduction efficacy indicated by luciferase expression (y-axis) measured by *in vivo* imaging system (IVIS) 24 h after transduction of solid tumor tissue with Ad5(GFP-Luc) in combination with vehicles (x-axis). Individual biological replicates (n=3), mean and 95 % CI are shown. No statistically significant differences were found between the group means, using Kruskal-Wallis test.
